# Epigenetics provides a new generation of oncogenes and tumour-suppressor genes

**DOI:** 10.1038/sj.bjc.6602918

**Published:** 2006-01-10

**Authors:** M Esteller

**Affiliations:** 1 Cancer Epigenetics Laboratory, 3rd Floor, Molecular Pathology Programme, Spanish National Cancer Centre (CNIO), Melchor Fernandez Almagro 3, 28029 Madrid, Spain

**Keywords:** epigenetics, DNA methylation, histones, chromatin

## Abstract

Cancer is nowadays recognised as a genetic and epigenetic disease. Much effort has been devoted in the last 30 years to the elucidation of the ‘classical’ oncogenes and tumour-suppressor genes involved in malignant cell transformation. However, since the acceptance that major disruption of DNA methylation, histone modification and chromatin compartments are a common hallmark of human cancer, epigenetics has come to the fore in cancer research. One piece is still missing from the story: are the epigenetic genes themselves driving forces on the road to tumorigenesis? We are in the early stages of finding the answer, and the data are beginning to appear: knockout mice defective in DNA methyltransferases, methyl-CpG-binding proteins and histone methyltransferases strongly affect the risk of cancer onset; somatic mutations, homozygous deletions and methylation-associated silencing of histone acetyltransferases, histone methyltransferases and chromatin remodelling factors are being found in human tumours; and the first cancer-prone families arising from germline mutations in epigenetic genes, such as hSNF5/INI1, have been described. Even more importantly, all these ‘new’ oncogenes and tumour-suppressor genes provide novel molecular targets for designed therapies, and the first DNA-demethylating agents and inhibitors of histone deacetylases are reaching the bedside of patients with haematological malignancies.

## CANCER AS AN EPIGENETIC DISEASE

Great effort has been directed in recent years towards understanding the establishment and relevance of aberrant epigenetic patterns in human tumours. For DNA methylation, it is known that two apparently contrasting phenomena coexist in the cancer cell: a profound loss of global 5-methylcytosine genomic content with discrete areas of dense hypermethylation (Jones and Baylin, 2002; Feinberg and Tycko, 2004; Esteller, 2005a). Overall hypomethylation takes place predominantly in DNA repetitive and endoparasitic sequences and has been linked to the generation of chromosomal instability (Feinberg and Tycko, 2004; Esteller, 2005a). On the other hand, hypermethylation occurs in the CpG islands located in the promoters of certain tumour-suppressor genes, such as p16^INK4a^, BRCA1 or hMLH, leading to gene silencing (Jones and Baylin, 2002; Esteller, 2005a).

Histones are another key player in epigenetics. Today they are recognised as having a primary role in the control of gene expression and chromatin structure and are partners closely involved with the DNA methylation machinery (Fraga and Esteller, 2005). Our knowledge of the behaviour of histones in cancer cells is slight compared with that of DNA methylation. We know that certain histone modifications participate in tumour-suppressor gene silencing, in conjunction with CpG island hypermethylation (Fahrner *et al*, 2002; Ballestar *et al*, 2003) or in its absence, such as the case of p21^WAF1^. Most importantly, we have recently demonstrated that human tumours undergo an overall loss of monoacetylation of lysine 16 and trimethylation of lysine 20 in the tail of histone H4. These two histone-modification losses can be considered as almost universal epigenetic markers of malignant transformation (Fraga *et al*, 2005), as has now been accepted for global DNA hypomethylation and CpG island hypermethylation. Certain histone acetylation and methylation marks may have prognostic value (Seligson *et al*, 2005).

Finally, we should not forget that DNA methylation and histone modifications are not isolated events, but occur in higher-order chromatin structure. Nucleosomes, formed by DNA wrapping around an octamer of histones, are the champions of that league. Multi-subunit complexes, such as those constituted by the SWI/SNF proteins, use the energy of ATP to mobilise nucleosomes and allow the access of the transcriptional machinery (Gibbons, 2005); or massive repressive complexes counteract SWI/SNF functions, as does the polycomb group gene family (Valk-Lingbeek *et al*, 2004). In the end, the impact on gene expression in cancer cells is massive and the normal epigenetic programming of the healthy cell becomes merely a faint memory.

**Figure 1:**
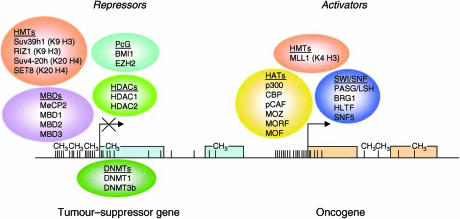
Epigenetic proteins that contribute to human tumorigenesis. Silencing of a classical tumours suppressor gene involves the recruitment of a transcriptional repressor machinery to the corresponding promoter CpG island, such as DNA methyltransferases (DNMT), methyl-CpG-binding proteins (MBD), histone methyltransferases for lysine 9 of histone H3 (HMT K9 H3), histone deacetylases (HDAC) and polycomb (PcGs) complexes. In the other side of the coin, the active expression of oncogenes in human cancer requires a potent transcriptional activation machinery, such as the one constituted by the chromatin-remodelling factors SWI/SNF, histone acetyltransferases (HATs) and histone methyltransferases for lysine 4 of histone H3 (HMT K4 H3).

Never the less, several pieces of the jigsaw are still missing. Are there molecular alterations acting as ‘dark horses’ behind this distorted epigenetic pattern? Could the epigenetic genes turn out to be the next generation of oncogenes and tumour-suppressor genes? Could these genes be targeted with new drugs? Let us review the data that is summarised in Figure 1 and Table 1.

## GENES MEDIATING THE DISRUPTION OF DNA METHYLATION

It has been known for a long time that there is more enzymatic DNA methyltransferase activity overall in tumours than in normal tissues (Esteller, 2005a, 2005b). This finding has been supported by the molecular characterisation of the genes encoding several DNA methyltranferases (DNMT1, DNMT3a, DNMT3b, DNMT3L and DNMT2), which has shown that the number of mRNA transcripts of DNMT1 (the classical methylation maintenance enzyme) and DNMT3b (the *de novo* methylation enzyme) are greater in several solid and haematological malignancies (Esteller, 2005a).

Balanced DNMT activity is most important for the prevention of cell transformation. The genetic disruption of two DNMTs, DNMT1 and DNMT3b, in a cancer cell line induces demethylation of all known hypermethylated tumour-suppressor genes (Rhee *et al*, 2002; Paz *et al*, 2003) and remarkably slow growth (Rhee *et al*, 2002). Somatic DNMT mutations have not so far been described in human tumours, although DNMT1 is located in 19p13.3, a region of common loss of heterozygosity in human tumours. DNMT3b germline mutation are responsible for the immunodeficiency centromeric instability-facial anomalies (ICF) syndrome, the cancer risk of which is not known, while no DNMT1 germline mutation in any genetic syndrome has so far been reported. Results obtained in mouse models again reflect the need for well-adjusted DNMT function to maintain cellular homeostasis: DNMT1 knockout mice are ‘protected’ against the development of colorectal adenomas when crossed with APC-deficient mice (Laird *et al*, 1995), but they are ‘prone’ to develop lymphomas in the context of mice susceptible to this type of neoplasia (Eden *et al*, 2003). These latter results may be explained if lymphomas rely predominantly on chromosomal instability dependent on genomic DNA hypomethylation, while colon tumours rely more on the CpG island methylation status of tumour-suppressor genes (Yamada *et al*, 2005).

Something has to read the DNA methylation markers. The most likely candidates are transcriptional repressors that have an appetite for methylated CpGs. These are the methyl-CpG-binding domain proteins (MBDs) (Esteller, 2005a). MBDs are important ‘translators’ between DNA methylation and histone modifier genes that establish a transcriptionally inactive chromatin environment. This family of proteins consists of five well-characterised members (MeCP2, MBD1, MBD2, MBD3 and MBD4) (Esteller, 2005a). MBD proteins are associated with hypermethylated CpG island promoters of tumour-suppressor genes and their transcriptional silencing (Esteller, 2005a), showing remarkable specificity *in vitro* (Fraga *et al*, 2003) and *in vivo* (Ballestar *et al*, 2003; Klose *et al*, 2005).

Individual loss of MeCP2, MBD1, MBD2, for which single knockout mice are viable, do not appear to affect tumour formation significantly (Esteller, 2005a), which suggests that the remaining MBDs may compensate for the function of the missing one. However, deficiency of MBD2 suppresses intestinal tumorigenesis in an Mbd2-knockout mouse derived from a lineage with an autosomal-dominantly inherited predisposition to multiple intestinal neoplasia (Min) (Sansom *et al*, 2003). We can hypothesise that the absence of MBD2 produces a ‘leak’ in the CpG island hypermethylation silencing of tumour-suppressor genes, thereby partially aborting aberrant cancer growth. This may not be universal for all tumour types and in this regard deficiency of MBD2 does not enhance lymphomagenesis in p53-deficient mice (Sansom *et al*, 2005). Expression analysis of MBD proteins in tumours has revealed increased overall levels associated with enhanced proliferation (Esteller, 2005a). Mutations in MBDs do occur in sporadic tumours, albeit rarely (Bader *et al*, 2003). MBD4 is an exception and is frequently targeted by inactivating frameshift mutations in microsatellite-unstable tumours (Riccio *et al*, 1999). However, we should keep in mind that MBD4 is unusual: it has a glycosylase domain that removes thymidine from T:G mismatches.

## GENES MEDIATING THE DISRUPTION OF HISTONE MODIFICATIONS

A first draft of an aberrant histone modification signature for human cancer has been produced (Fraga *et al*, 2005; Fraga and Esteller, 2005; Seligson *et al*, 2005). The next task is to identify the molecules involved in its establishment: histone acetyltransferases (HATs), histone methyltransferases (HMTs) and histone deacetylases (HDACs).

With respect to histone acetylation, we have found a loss of recruitment of a family of the specific K-16 HATs MOZ, MOF and MORF to DNA-repetitive sequences in cancer cells (Fraga *et al*, 2005). These HATs are already altered in leukaemias by the generation of fusion proteins such as MOZ-CBP and MORF-CBP that are also associated with significant global losses of acetylation of K16-H4 (Fraga *et al*, 2005). What makes the case even more interesting is that the other partner of the fusion protein generated is usually CBP or p300, two other HATs with numerous substrates, but that do not act on lysine 16 of H4 (Fraga and Esteller, 2005). A tumour-suppressor role for these global HATs has been strongly suggested from many sources: CBP, p300 and pCAF somatic mutations have been described in primary human tumours (Gayther *et al*, 2000; Ozdag *et al*, 2002; Ionov *et al*, 2004; Kishimoto *et al*, 2005); patients with Rubinstein–Taybi syndrome, caused by germline mutations in the CBP gene, have an increased tendency to develop tumours at an early age (Gibbons, 2005); and CBP heterozygous mice develop haematological tumours (Gibbons, 2005).

In the case of HDAC, the deacetylation of K16-H4 seems to be particularly closely regulated: in yeast, Sir2 deacetylates this residue, and its human homologue, Sirtuin 1 (SIRT1), also deacetylates the tumour-suppressor protein p53, thereby establishing another link with cancer. We can view the picture from this angle and hypothesise that there is increased recruitment of SIRT1 to the K16-H4 position in repeat DNA sequences in the transformed cell. In this regard, overexpression of SIRT1 is observed in leukemia cells (Bradbury *et al*, 2005). For HDACs with a broader deacetylation specificity than SIRT1, such as HDAC1 and HDAC2, no somatic mutations in tumours have been described, but a dysregulated expression seems to be a common feature of human neoplasia (Gibbons, 2005).

A similar scenario could be proposed for the trimethylation of lysine 20 of H4 (Fraga *et al*, 2005). This reaction is catalysed by the HMTs Suv4-20h1 and Suv4-20h2, in addition to PR/SET7-SET8 (Fraga and Esteller, 2005). These enzymes can be targets for disruption in cancer cells, as occurs with another HMT, MLL1, which is translocated to multiple partners in haematological malignancies (Fraga and Esteller, 2005). The loss of trimethylation of histone H4 in cancer cells might be a consequence of a deficiency of these enzymatic activities in tumours, and/or a lack of recruitment to heterochromatic regions, where most of the histones containing such modifications are localised. We should keep in mind that mice deficient in SUV39H1 HMTs (which target lysine 9 of histone H3) have increased chromosomal instability and tumour risk (Peters *et al*, 2001) and another K9-H3 histone methyltransferase, RIZ1, undergoes CpG island hypermethylation-associated silencing in many tumour types (Esteller, 2005a). Lack of HMTs specific to K20-H4 has not yet been described in tumours, but all these issues can now be addressed with new tools, such as specific antibodies against Suv4-20h (Fraga *et al*, 2005).

## GENES MEDIATING THE DISRUPTION OF CHROMATIN REMODELLING

Polycomb-group (PcG) and trithorax-group (trxG) genes are epigenetic silencers and activators for gene transcription, respectively (Valk-Lingbeek *et al*, 2004; Raaphorst, 2005). Both are part of multitask protein complexes including HDAC and HMTs activities. In the case of the human homologue of trithorax, MLL, I have already mentioned the existence of translocations that generate transforming fusion proteins (Gibbons, 2005). For PcG in human there are two complexes, polycomb repressive complex 1 (PRC1) and 2 (PRC2), which contain the BMI1 and enhancer of zeste homologue 2 (EZH2) oncogenes, respectively (Valk-Lingbeek *et al*, 2004; Raaphorst, 2005). BMI1 downregulates the expression of tumour-suppressor genes, such as p16^INK4a^ and p14^ARF^ (Valk-Lingbeek *et al*, 2004), transgenic mice have a high predisposition to lymphomagenesis, subtypes of human lymphomas harbour BMI1 gene amplification and overexpression and it induces cell immortalisation (Valk-Lingbeek *et al*, 2004; Raaphorst, 2005). EZH2 undergoes gene amplification in several tumour types (Bracken *et al*, 2003), it is overexpressed in prostate and breast cancers, and its downregulation leads to growth inhibition (Raaphorst, 2005).

Another powerful epigenetic repressor family of chromatin-remodelling factors is the Tudor domain ‘Royal Family’ (Hughes-Davies *et al*, 2003). Two of its members, HP1 and BS69, bind to EMSY. The latter gene is a critical component in breast cancer development in the BRCA2 pathway. Most importantly, EMSY behaves as an oncogene in mammary neoplasm, where it shows gene amplification (Hughes-Davies *et al*, 2003). The list of targeted epigenetic repressors also includes Breast Cancer Metastasis Suppressor 1 (BRMS1), which is able to inhibit metastasis without ‘touching’ tumorigenesis itself (Meehan and Welch, 2003). BRMS1 joins the mSin3a HDAC complexes to silence the targeted genes (Meehan and Welch, 2003). An opposing force to BRMS1 is the group of metastasis-associated genes (MTAs). MTA1, MTA2, and MTA3 are components of the nucleosome remodelling and deacetylation complex, as a part of the NuRD complex. Metastasis-associated gene 1 overexpression is associated with the most malignant behaviour in several human tumours. Metastasis-associated gene 3 has a predominant role in lymphoma and breast cancer, in the latter leading to aberrant expression of the transcriptional repressor Snail and loss of expression of the cell-adhesion molecule E-cadherin, an event associated with invasive growth of breast cancers (Fujita *et al*, 2003).

The best-recognised chromatin remodelling ‘force’ is the SWI/SNF family of ATP-hydrolysing enzymes (Gibbons, 2005). These are multiprotein complexes, as we have seen in the case of the PcG, but in this case with transcriptional activation properties. Among the many members of the SWI/SNF family, four are involved with cancer development: PASG/LSH, BRG1, HLTF and SNF5. Potential mutations of PASG/LSH have been identified in leukaemia (Lee *et al*, 2000); BRG1 genetic alterations occur in several tumour types and the reintroduction of the gene inhibits cell growth (Medina *et al*, 2005); helicase-like transcription factor (HLTF) undergoes methylation-associated silencing in different neoplasms (Esteller, 2005a); and SNF5 shows inactivating somatic and germline mutations in malignant rhabdoid tumours (Imbalzano and Jones, 2005).

## TARGETING EPIGENETIC GENES IN CANCER THERAPY

The ‘holy grail’ of current research in cancer therapy is the design of specific drugs against molecular alterations found only in the transformed cell, such as mutations in oncogenes. Epigenetics can offer many new targets for this approach. However, only two types of epigenetic drugs, neither of which is very specific, have nowadays a real impact: DNA-demethylating agents and histone deacetylase inhibitors (HADCis) (Villar-Garea and Esteller, 2004; Esteller, 2005b). We have to be patient with other potential epigenetic drugs, such as histone acetyltransferase inhibitors, including anacardic acid, curcumin, and peptide CoA conjugates; in addition to undisclosed histone methyltransferase inhibitors or those HDACis that are specific for SIRT1 (class III HDAC), such as nicotinamide and splitomycin.

Of the class of DNA-demethylating agents, the first drug used to inhibit DNA methylation was 5-azacytidine (Vidaza). This substance causes covalent arrest of DNMTs, resulting in cytotoxicity. 5-Azacytidine was tested for its usefulness as an antileukaemic drug before its demethylating activity was known (Esteller, 2005b). The analogue 5-aza-2′-deoxycytidine (Decitabine) is one of the most commonly used demethylating drugs in assays with cultured cells. Zebularine is another recently developed cytidine analogue (Yoo *et al*, 2004). It forms a covalent complex with DNA methyltransferases (Yoo *et al*, 2004). Furthermore, zebularine has also shown promising antitumoral effects in xenografts (Yoo *et al*, 2004) and thymic lymphomas (Herranz *et al*, 2005) in mice. Perhaps the most interesting feature of this DNA-demethylating agent is that it is chemically stable and of low toxicity (Yoo *et al*, 2004; Herranz *et al*, 2005), and can be taken orally. It is in the field of haematological malignancies that DNA-demethylating agents have had their greatest success so far, especially in high-risk myelodysplastic syndrome using 5-aza-2-deoxycytidine (Esteller, 2005b). In 2004, the FDA approved the use of 5-azacytidine (Vidaza) for the treatment of all myelodysplastic syndrome subtypes.

On the other hand, naturally occurring and synthetic HDACis are also the focus of interest because of their great potential utility against cancer. Overall, HDACis manifest a wide range of activities against all HDACs. These compounds can be classified into the following groups according to their chemical nature: hydroxamic acids, such as trichostatin A, SAHA, PXD101 and NVP-LAQ-824; carboxylic acids, such as sodium valproate and butyrate; benzamides, such as MS-272 and others, including trapoxins and FK228 (Villar-Garea and Esteller, 2004). It is believed that the anticancer effects of HDACis are mediated by the reactivation of the expression of tumour-suppressor genes. However, the treatment of cancer cell lines with HDACis has pleiotropic effects inducing differentiation, cell-cycle arrest and apoptosis. In this regard, the observation that cancer cells have lost monoacetylated lysine 16 histone H4 (Fraga *et al*, 2005) implies a new molecular pathway that may explain the beneficial effects of HDAC inhibitors because these compounds may promote the restoration of normal histone H4 acetylation levels in the whole cell, restoring the normal chromatin status of repetitive DNA sequences (Fraga *et al*, 2005). It is clear from *in vitro*, preclinical studies and ongoing clinical trials that HDACis have enormous potential as anticancer drugs. In this regard, SAHA may soon be approved for the treatment of cutaneous lymphoma.

## CONCLUSIONS

Cancer is a genetic and epigenetic disease. We cannot study and explore one field and ignore the other. The overall disruption of the epigenetic landscape is the most common feature of all human tumours. This includes global loss of genomic DNA methylation, local CpG island hypermethylation-associated gene silencing and a characteristic histone modification pattern. Stochastic and selective forces drive the whole process, and in the same way as in the cell cycle, apoptosis and DNA repair genes are targeted, genes involved in DNA methylation, histone modification and chromatin remodelling also become disrupted. Some of these will act as oncogenes, others as tumour-suppressor genes. Some will be altered by genetic lesions, others by epigenetic lesions. Ultimately, we will have to find better drugs to combat both processes.

## Figures and Tables

**Table 1:** A selected list of epigenetic genes disrupted in human cancer and additional potential candidates

**Gene**	**Function**	**Alteration**	**Tumour profile**
DNMT1	DNA methyltransferase	Overexpression	Multiple types
DNMT3b	DNA methyltransferase	Overexpression	Multiple types
MeCP2	Methyl-CpG-binding protein	Overexpression, rare mutations	Multiple types
MBD2	Methyl-CpG-binding protein	Overexpression, rare mutations	Multiple types
MBD1	Methyl-CpG-binding protein	Overexpression, rare mutations	Multiple types
MBD3	Methyl-CpG-binding protein	Overexpression, rare mutations	Multiple types
MBD4	Methyl-CpG-binding protein	Inactivating mutations in MSI+	Colon, stomach, endometrium
p300	Histone acetyltransferase	Mutations	Colon, stomach, endometrium
CBP	Histone acetyltransferase	Mutations, homozygous deletions	Colon, stomach, endometrium, lung
pCAF	Histone acetyltransferase	Rare mutations	Colon
MOZ	Histone acetyltransferase	Translocations	Haematological malignancies
MORF	Histone acetyltransferase	Translocations	Haematological malignancies
MOF	Histone acetyltransferase	Unknown	Unknown
HDAC1	Histone deacetylase	Imbalanced expression	Multiple types
HDAC2	Histone deacetylase	Imbalanced expression	Multiple types
SIRT1	Histone deacetylase	Unknown	Unknown
SUV39H1	Histone methyltransferase	Unknown	Unknown
Suv4-20h	Histone methyltransferase	Unknown	Unknown
PR/SET7,8	Histone methyltransferase	Unknown	Unknown
RIZ1	Histone methyltransferase	CpG island hypermethylation, mutation	Multiple types
MLL1	Histone methyltransferase	Translocation	Haematological malignancies
BMI1	Polycomb-group protein	Gene amplification, overexpression	Haematological malignancies, brain
EZH2	Polycomb-group protein	Gene amplification, overexpression	Multiple types
EMSY	Chromatin-remodelling factor	Gene amplification, overexpression	Breast
BRMS1	Chromatin-remodelling factor	Loss of expression	Breast
MTA1	Chromatin-remodelling factor	Overexpression	Breast, haematological malignancies
MTA3	Chromatin-remodelling factor	Overexpression	Breast, haematological malignancies
PASG/LSH	SWI/SNF family protein	Mutation	Haematological malignancies
BRG1	SWI/SNF family protein	Homozygous deletion, mutation	Lung
HLTF	SWI/SNF family protein	CpG island hypermethylation	Multiple types
SNF5	SWI/SNF family protein	Somatic and germline mutations	Rhabdoid tumours

MSI+=microsatellite instable tumours.
